# Re-evaluation of soluble APP-α and APP-β in cerebrospinal fluid as potential biomarkers for early diagnosis of dementia disorders

**DOI:** 10.1186/s40364-017-0108-5

**Published:** 2017-09-22

**Authors:** Wataru Araki, Kotaro Hattori, Kazutomi Kanemaru, Yuma Yokoi, Yoshie Omachi, Harumasa Takano, Masuhiro Sakata, Sumiko Yoshida, Tadashi Tsukamoto, Miho Murata, Yuko Saito, Hiroshi Kunugi, Yu-ichi Goto, Utako Nagaoka, Masahiro Nagao, Takashi Komori, Kunimasa Arima, Kenji Ishii, Shigeo Murayama, Hiroshi Matsuda, Hisateru Tachimori, Yumiko M. Araki, Hidehiro Mizusawa

**Affiliations:** 1 Department of Demyelinating Disease and Aging, National Institute of Neuroscience, National Center of Neurology and Psychiatry (NCNP), 4-1-1 Ogawahigashi, Kodaira, Tokyo, 187-8502 Japan; 2 Medical Genome Center, NCNP, Tokyo, Japan; 3 Tokyo Metropolitan Geriatric Hospital and Institute of Gerontology, Tokyo, Japan; 4 National Center Hospital, NCNP, Tokyo, Japan; 5 Department of Mental Disorder Research, National Institute of Neuroscience, NCNP, Tokyo, Japan; 6 Tokyo Metropolitan Neurological Hospital, Tokyo, Japan; 7 Komoro Kogen Hospital, Komoro, Japan; 8 Integrative Brain Imaging Center, NCNP, Tokyo, Japan; 9 Department of Mental Health Policy and Evaluation, National Institute of Mental Health, NCNP, Tokyo, Japan; 10 Department of Psychiatry and Behavioral Science, Graduate School of Medicine, Juntendo University, Tokyo, Japan

**Keywords:** Alzheimer’s disease, Biomarker, Cerebrospinal fluid, Mild cognitive impairment, Soluble amyloid precursor protein, Tau

## Abstract

**Background:**

Because soluble (or secreted) amyloid precursor protein-β (sAPPβ) and -α (sAPPα) possibly reflect pathological features of Alzheimer’s disease (AD), they are potential biomarker candidates for dementia disorders, including AD and mild cognitive impairment (MCI) due to AD (MCI-AD). However, controversial results have been reported regarding their alterations in the cerebrospinal fluid (CSF) of AD and MCI-AD patients. In this study, we re-assessed the utility of sAPPα and sAPPβ in CSF as diagnostic biomarkers of dementia disorders.

**Methods:**

We used a modified and sensitive detection method to analyze sAPPs levels in CSF in four groups of patients: AD (*N* = 33), MCI-AD (*N* = 17), non-AD dementia (*N* = 27), and disease controls (*N* = 19). Phosphorylated tau (p-tau), total tau, and Aβ42 were also analyzed using standard methods.

**Results:**

A strong correlation was observed between sAPPα and sAPPβ, consistent with previous reports. Both sAPPα and sAPPβ were highly correlated with p-tau and total tau, suggesting that sAPPs possibly reflect neuropathological changes in the brain. Levels of sAPPα were significantly higher in MCI-AD cases compared with non-AD and disease control cases, and those of sAPPβ were also significantly higher in MCI-AD and AD cases relative to other cases. A logistic regression analysis indicated that sAPPα and sAPPβ have good discriminative power for the diagnosis of MCI-AD.

**Conclusions:**

Our findings collectively suggest that both sAPPs are pathologically relevant and potentially useful biomarkers for early and accurate diagnosis of dementia disorders. We also suggest that careful measurement is important in assessing the diagnostic utility of CSF sAPPs.

**Electronic supplementary material:**

The online version of this article (10.1186/s40364-017-0108-5) contains supplementary material, which is available to authorized users.

## Background

Alzheimer’s disease (AD) is a neurodegenerative disorder neuropathologically characterized by senile plaques and neurofibrillary tangles, which are mainly composed of amyloid β-protein (Aβ) and phosphorylated tau protein, respectively [1]. Recent clinical studies have revealed that the neuropathology of AD starts many years before symptom onset and is apparent at the stage of mild cognitive impairment (MCI) due to AD (MCI-AD) or prodromal AD [2]. On the other hand, various disease-modifying treatments are being developed and tested in clinical trials [3, 4]. Because of these clinical features and ongoing therapeutic development, it has become increasingly critical to accurately diagnose dementia disorders at earlier stages [2].

Among the diagnostic biomarkers of dementia disorders, cerebrospinal fluid (CSF) biomarkers are regarded as particularly reliable. One reason is that CSF directly interacts with the extracellular space in the brain and thus reflects the associated biochemical and pathological changes [5]. In fact, Aβ42 and tau (total tau and phosphorylated tau) are widely accepted as core CSF biomarkers for the diagnosis of AD dementia [5–8]. Although these biomarkers are highly useful and now included in the diagnostic criteria, they still have some limitations, such as inter- and intra-laboratory variabilities and substantial overlap with other forms of dementia [5, 8–10]. Furthermore, no biomarkers are currently available that are specific for MCI-AD. Thus, it is generally thought that addition of other biomarkers could improve the accuracy of early diagnosis of dementia disorders [5].

Aβ is derived from proteolytic processing of amyloid precursor protein (APP). Proteolysis of APP by β-secretase (BACE1) generates soluble (or secreted) APP-β (sAPPβ) and β-C-terminal fragment (β-CTF), and γ-secretase cleavage of the latter yields Aβ. Alternative processing of APP by α-secretases, mainly ADAM10 (a disintegrin and metallopeptidase domain 10), generates sAPPα and α-CTF [11]. In AD brains, expression levels of BACE1 are increased, potentially influencing sAPPβ levels [12]. Thus, sAPPβ likely reflects pathological changes in BACE1. Similarly, generation of sAPPα may be altered under pathological conditions. Therefore, both sAPPα and sAPPβ have been regarded as potential biomarkers for dementia disorders; however, controversial results have been reported regarding their alterations in CSF of patients with AD or MCI-AD [13]. Moreover, CSF sAPPs appear to be useful biomarkers for monitoring effects of disease-modifying agents such as BACE1 inhibitors [13, 14].

In this study, we sought to re-assess the utility of sAPPα and sAPPβ in CSF as reliable diagnostic biomarkers for AD and/or MCI-AD. For this purpose, we used sensitive modified methods for detection of sAPPs. Our present findings support the utility of sAPPα and sAPPβ for early diagnosis of dementia disorders.

## Methods

### Subjects

This study was made possible by the collaboration of three institutions/hospitals located in Tokyo, Japan: National Center of Neurology and Psychiatry (NCNP), Tokyo Metropolitan Geriatric Hospital (TMGH), and Tokyo Metropolitan Neurological Hospital (TMNH), and was conducted with approval of the ethics committee of the respective institutions/hospitals. CSF samples used in this study were collected between 2008 and 2016 with informed consent of participants. The clinical diagnostic protocol included a neurological examination, neuropsychological tests and evaluations (MMSE, HDS-R [Hasegawa’s Dementia Scale-Revised], CDR [Clinical Dementia Rating], etc.), brain-imaging tests (MRI and SPECT), and CSF biomarkers (Aβ1–42, total tau, and tau phosphorylated at Thr181 [p-tau]). Only selected patients underwent positron emission tomography (PET) studies (i.e., FDG-PET and Pittsburgh Compound B [PiB]-PET); e.g., [PiB]-PET was performed in 65% of MCI-AD subjects. Diagnoses were made by experienced neuropsychiatrists or neurologists. Patients in the study were divided into four groups: AD, MCI-AD, non-AD dementias (non-AD), and non-dementia neurological disorders (disease controls) (Table 1). Patients with MCI with a cognitive syndrome unlikely due to AD (MCI-others) were excluded from this study. All patients with AD and MCI-AD met core clinical criteria proposed by the National Institute on Aging and the Alzheimer’s Association (NIA-AA) workgroup [15, 16]. Non-AD dementias included frontotemporal dementias (FTD), dementia with Lewy bodies (DLB), corticobasal syndrome (CBS), and progressive supranuclear palsy (PSP); these conditions were diagnosed based on characteristic clinical symptoms, the findings of brain-imaging tests, and other tests useful for differential diagnosis [17, 18]. Disease controls included spinocerebellar ataxia, multiple system atrophy, Parkinson’s disease, brain tumor, epilepsy, normal pressure hydrocephalus, mood disorders, psychosis, and old cerebral hemorrhage.

**Table 1:** Demographics and biomarker results of the study cohort

	Number of subjects				Age	Gender	sAPPα	sAPPβ	p-tau	Aβ42
Groups	Total	NCNP	TMGH	TMNH		(M/F)	(ng/ml)	(ng/ml)	(pg/ml)	(pg/ml)
AD	33	21	3	9	75.5 ± 1.5	15/18	320.6 ± 22.6	594.9 ± 39.7	89.1 ± 5.8	677.8 ± 34.0
MCI-AD	17	7	7	3	70.6 ± 2.1	7/10	468.0 ± 66.4	785.4 ± 101.2	91.7 ± 9.5	562.0 ± 60.8
Non-AD	27	18	3	6	72.6 ± 1.6	14/13	235.5 ± 24.9	417.6 ± 33.6	43.9 ± 3.8	844.3 ± 55.2
Dis. control	19	16	0	3	67.5 ± 1.9	12/7	222.8 ± 25.0	383.6 ± 34.3	36.1 ± 2.7	1013.0 ± 71.2

Data of statistical analyses are described in Fig. 3 and the text

The demographic data of patients are shown in Table 1. There were no significant differences in the mean age of the patients among AD, MCI-AD, non-AD dementia, and disease control groups, except for that between AD and disease controls patients (*p* < 0.05).

### CSF analyses

CSF was sampled by lumber puncture at the L3/L4 intervertebral space, collected in polypropylene tubes, and stored in polypropylene cryotubes at −80 °C. Most CSF samples at NCNP were obtained from the NCNP Biobank. Aβ1–42 was assayed by using an INNOTEST β-AMYLOID_(1–42)_ kit (Fujirebio, Gent, Belgium); total tau and p-tau were assayed using a Fino Scholar hTAU kit (Nipro, Osaka, Japan) and INNOTEST PHOSPHO-TAU_(181P)_ kit (Fujirebio), respectively, according to the manufacturers’ instructions. Measurement of these three biomarkers was performed at NCNP for CSF samples from NCNP and TMNH, and at TMGH for samples from TMGH. Only Aβ42 and p-tau data were included for TMGH patients because of some inconsistencies in the measurement of total tau.

sAPPα and sAPPβ in CSF were principally measured at the National Institute of Neuroscience, NCNP, using commercial ELISA kits (Human sAPPα Assay Kit, and Human sAPPβ-w Assay Kit; IBL, Gunma, Japan) with modifications to enhance the sensitivity of detection and to minimize the amount of CSF samples required. Typically, CSF samples were diluted 1:20 for sAPPα and 1:25 for sAPPβ (1:30 in some cases) with phosphate-buffered saline (PBS), and 100 μl of each was applied to duplicate wells of strips on a 96-well plate, precoated with anti-sAPPα or anti-sAPPβ antibody. Standards were prepared as described in the kit manual and applied as described above. After incubation at 4 °C overnight, plates were rinsed seven times with wash buffer, and then 100 μl of labeled antibody (HRP-conjugated anti-human APP), diluted 1:30 in Can Get Signal Immunoreaction Enhancer Solution (Toyobo, Osaka, Japan), was added to each well. After incubation at 4 °C for 1 h, plates were rinsed as above, and 100 μl of TMB solution from a TMB Microwell Peroxidase Substrate System kit (KPL, Gaithersburg, MD, USA) was added to each well. The reaction was then terminated by adding 100 μl of stop solution (1 M phosphoric acid), and absorbance in wells was measured at 450 nm using a plate reader. The concentrations of markers in samples were calculated by reference to standard curves. Significant differences were noted between the patterns of typical standard curves obtained using our modified method and those obtained using the original method (Additional file 1: Figure S1). An analysis of the same samples using the original and modified method showed that both methods yielded equivalent concentrations of sAPPα, whereas the original method yielded lower sAPPβ concentrations (~80%) compared with the modified method. These data imply that the original method underestimates the amount of sAPPβ.

### Statistical analysis

Possible correlations between concentrations of biomarkers of interest were evaluated by calculating Pearson’s product moment correlation or Spearman’s rank-order correlation coefficients.

To test differences in the concentrations of sAPPα, sAPPβ, and p-tau between studied groups (i.e. AD, MCI-AD, non-AD dementia, and disease control), logarithmic transformation of the data was performed, and the analysis of covariance (ANCOVA) was used, controlling for the effects of age. If a significant difference was found by the ANCOVA, pairwise comparison was performed with the use of the Bonferroni correction for multiple testing. The Kruskal-Wallis test was used for Aβ42 to detect significant differences in its concentrations between studied groups, since the variances of the studied groups did not seem to be equal in Aβ42.

To evaluate the diagnostic power of the biomarkers (sAPPα, sAPPβ, p-tau, and the combination of sAPPα and sAPPβ) for MCI-AD versus other studied groups (AD, non-AD dementia, and disease control), the logistic regression analyses were performed. The receiver operating characteristic (ROC) curves were drawn, and area under the curve (AUC) and its 95% confidence interval was calculated for each biomarker. Similar analyses were performed to evaluate the diagnostic power of the biomarkers for the diagnosis of AD and MCI-AD versus other groups.

All *P* values are two–tailed and those under 0.05 are considered statistically significant. Statistical analyses were performed using SPSS Statistics 24 (Japanese version; IBM Japan, Tokyo, Japan) and R [19].

## Results

### Correlation between sAPPα and sAPPβ

**Fig. 1:**
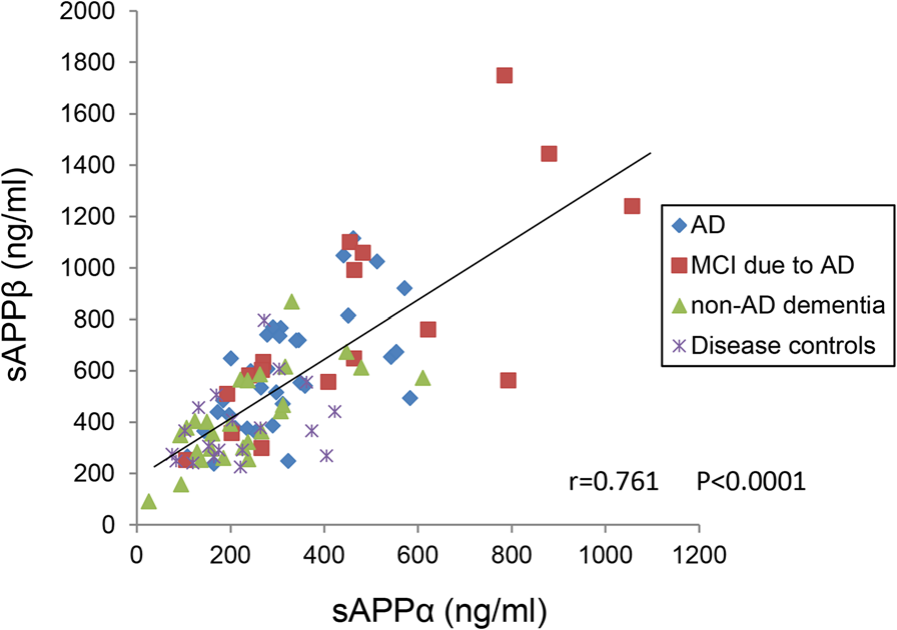
Scatterplots showing the correlation between sAPPα and sAPPβ. sAPPα and sAPPβ were measured in CSF in all participants (AD, MCI-AD, non-AD, and disease control). Possible correlations between these two biomarkers were evaluated by calculating a Pearson correlation coefficient

We first examined the concentrations of sAPPα and sAPPβ in CSF samples from the four groups of patients: AD, MCI-AD, non-AD dementia, and disease controls. As shown in Fig. 1, an analysis of the relationship between sAPPα and sAPPβ among all participants showed a strong positive correlation between sAPPα and sAPPβ levels (*r* = 0.757, *p* < 0.0001), consistent with previous studies [20–23]. Plotting the four groups separately revealed notably greater values of both sAPPα and sAPPβ among MCI-AD subjects compared with other groups (Fig. 1). A similar positive correlation between sAPPα and sAPPβ was observed among AD and MCI-AD groups (*r* = 0.734, *p* < 0.0001) as well as non-AD and disease control groups (*r* = 0.585, *p* < 0.0001). There was no association of age with levels of sAPPα (*r* = 0.046) or sAPPβ (*r* = −0.008) among all patients. Analyses of all patients, AD and MCI-AD groups, and the AD group revealed no statistical difference in sAPPα or sAPPβ levels between male and female patients.

### Correlation between sAPPs and tau or Aβ42

**Fig. 2:**
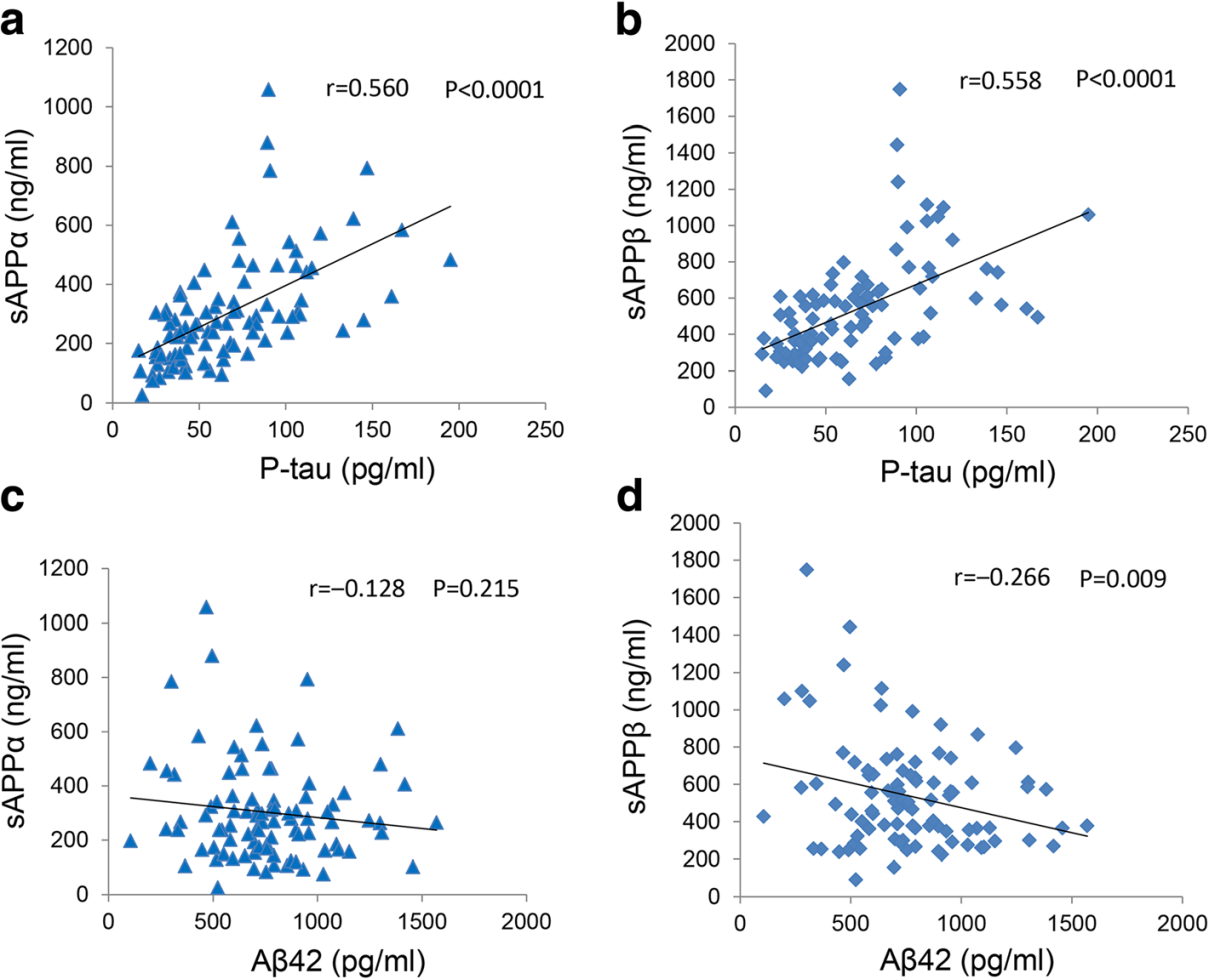
Correlation between p-tau and sAPPα or sAPPβ. Scatterplots, performed as in Fig. 1, show correlations between p-tau and sAPPα (**a**), p-tau and sAPPβ (**b**), Aβ42 and sAPPα (**c**), and Aβ42 and sAPPβ (**d**) among all participants

Next, we evaluated the correlation between sAPPs and tau (p-tau and total tau). Interestingly, we observed moderate positive correlations between p-tau and sAPPα (*r* = 0.591, *p* < 0.0001) and between p-tau and sAPPβ (*r* = 0.569, *p* < 0.0001) among all cases (Fig. 2a, b). Similar positive correlations were observed between p-tau and sAPPα and p-tau and sAPPβ among AD and MCI-AD groups (sAPPα: *r* = 0.434, *p* = 0.002; sAPPβ: *r* = 0.336, *p* = 0.017) and among other groups (sAPPα: *r* = 0.536, *p* = 0.0001; sAPPβ: *r* = 0.529, *p* = 0.0002), suggesting that differences in brain pathology do not considerably influence the association between p-tau and sAPPs. Similarly, both sAPPα (*r* = 0.628, *p* < 0.0001) and sAPPβ (*r* = 0.540, *p* < 0.0001) were positively correlated with total tau among all patients (Additional file 2: Figure S2) as well as among AD and MCI-AD groups (sAPPα: *r* = 0.577, *p* = 0.0001; sAPPβ: *r* = 0.359, *p* = 0.023) and other groups (sAPPα: *r* = 0.391, *p* = 0.01; sAPPβ: *r* = 0.349, *p* = 0.024). In contrast, there was no correlation between Aβ42 and sAPPα (*r* = −0.128, *p* = 0.215) among all patients, and there was a weak, but significant, negative correlation between Aβ42 and sAPPβ (*r* = −0.266, *p* = 0.009) (Fig. 2c, d). No correlation was observed between Aβ42 and sAPPα or sAPPβ among AD and MCI-AD groups or other groups.

### Diagnostic strength

**Fig. 3:**
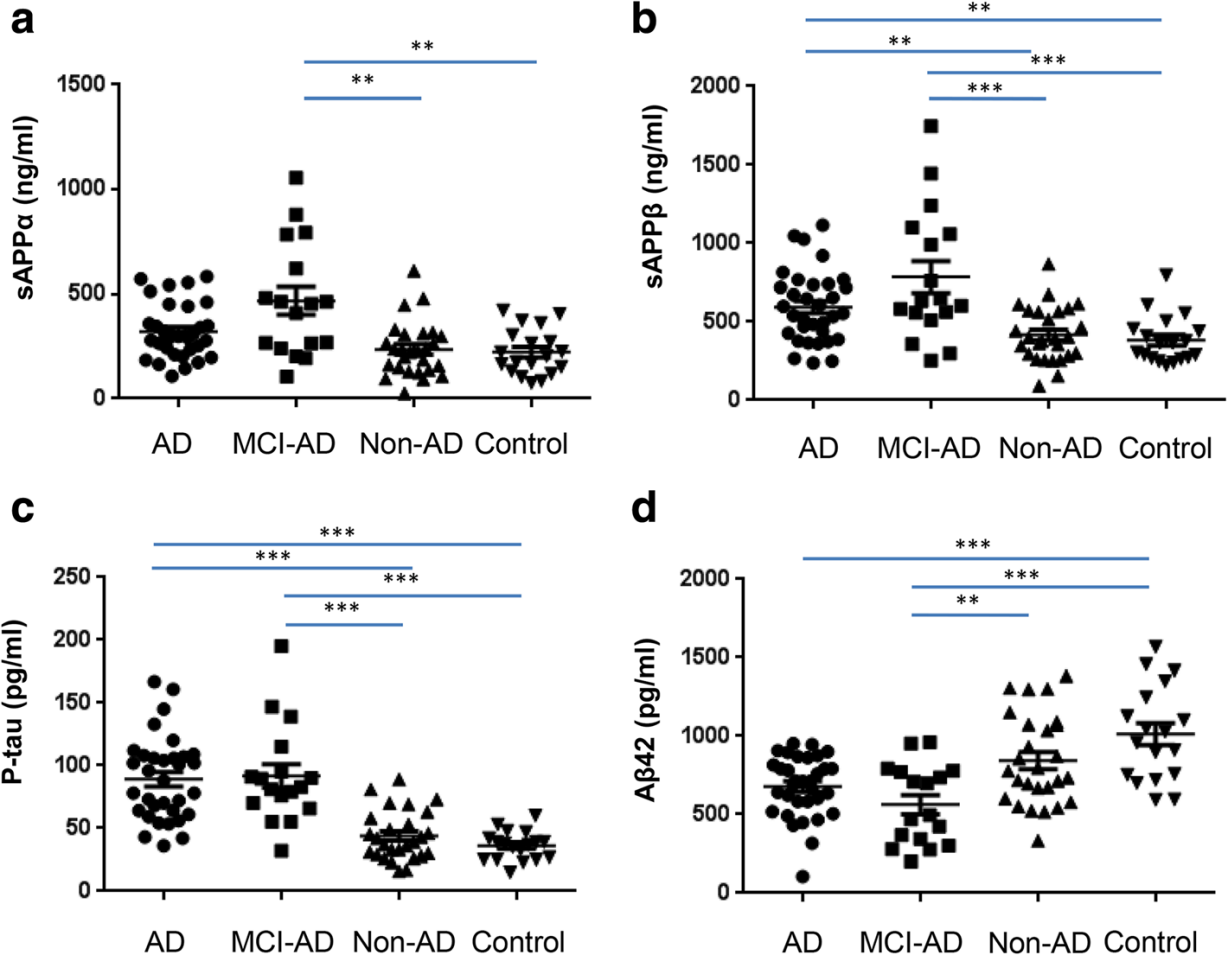
Levels of sAPPα (**a**), sAPPβ (**b**), p-tau (**c**), and Aβ42 (**d**) across the four groups of patients (AD, MCI-AD, non-AD, and disease control). Significant differences were analyzed by the methods described in Materials and Methods (*p < 0.05, **p < 0.01, ***p < 0.001)

To assess the diagnostic strengths of sAPPα and sAPPβ, we compared their concentrations among the four groups and evaluated their diagnostic utility in comparison with p-tau and Aβ42. We found that sAPPα levels in the MCI-AD group were significantly increased compared with the non-AD dementia and disease control groups, but were not significantly different from those in the AD group (Fig. 3a). Levels of sAPPβ were significantly increased in the MCI-AD and AD groups compared with the non-AD dementia and disease controls groups (Fig. 3b). As established diagnostic markers of AD and MCI-AD, p-tau levels were significantly elevated in both the AD and MCI-AD groups compared with the non-AD dementia and disease control groups, and Aβ42 levels were reduced in the AD and MCI-AD groups compared with the disease control group (Fig. 3c, d). sAPPβ showed a trend similar to that of p-tau.

**Fig. 4:**
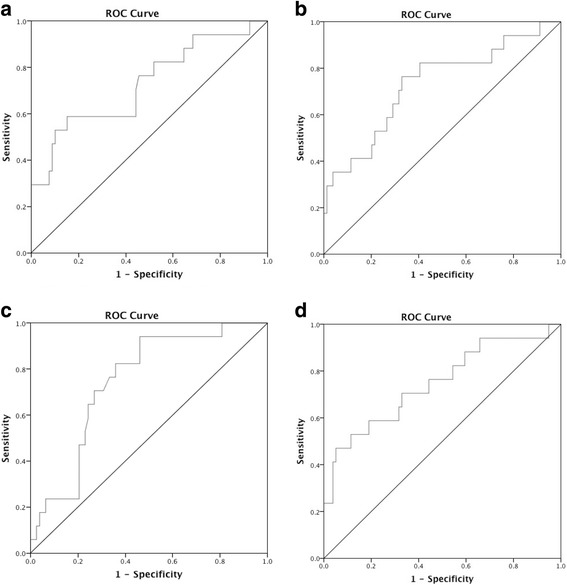
ROC curves indicating the discriminating ability of sAPPα (**a**), sAPPβ (**b**), and p-tau (**c**) in MCI-AD versus other groups (AD, MCI-O, non-AD, and disease control). For sAPPα, area under the curve (AUC) = 0.729 [Asymptotic 95% Confidence Interval: 0.584–0.873] and the appropriate cut off value is sAPPα = 250, with sensitivity = 0.765 and specificity = 0.544. For sAPPβ, AUC = 0.730 [0.589–0.872] and the appropriate cut off value is sAPPβ = 586, with sensitivity = 0.824 and specificity = 0.595. For p-tau, AUC = 0.745 [0.631–0.858] and the appropriate cut off value is p-tau = 66, with sensitivity = 0.824 and specificity = 0.641. (**d**) ROC curve for the combination of sAPPβ and sAPPα. AUC = 0.747 [0.605–0.889]

Logistic regression analyses were used to evaluate the diagnostic power of the biomarkers for MCI-AD versus other groups (AD, non-AD dementia, disease control). sAPPα, sAPPβ, and p-tau could each differentiate MCI-AD from other groups with AUC values of 0.729 (95% confidence interval [CI] = 0.584–0.873), 0.730 (95% CI = 0.589–0.872), and 0.745 (95% CI = 0.631–0.858), respectively (Fig. 4a-c). The combination of sAPPα and sAPPβ showed only slightly higher discriminatory power with an AUC of 0.747 (95% CI = 0.605–0.889) (Fig. 4d). In addition, the combination of sAPPα, sAPPβ, and p-tau and that of sAPPα, sAPPβ, p-tau, and Aβ42 altogether yielded discriminatory power with AUC values of 0.759 (95% CI = 0.621–0.898) and 0.836 (95% CI = 0.742–0.931), respectively (data not shown). Similar analyses were performed to evaluate the diagnostic power for the diagnosis of AD and MCI-AD versus other groups (non-AD dementia and disease control). sAPPα, sAPPβ, and p-tau could each differentiate AD and MCI-AD from other groups with AUC values of 0.736 (95% CI = 0.637–0.835), 0.766 (95% CI = 0.672–0.861), and 0.918 (95% CI = 0.864–0.972), respectively (Additional file 3: Figure S3).

## Discussion

In this study, we re-evaluated the utility of sAPPα and sAPPβ in CSF as potential biomarkers for dementia disorders, using a modified method to measure sAPPs. We demonstrated that sAPPβ levels were increased in both MCI-AD and AD groups compared with non-AD and control groups, whereas sAPPα levels were elevated only in the MCI-AD group compared with other groups. Furthermore, both sAPPα and sAPPβ levels were strongly correlated with p-tau and total tau levels. Accordingly, our data suggest that measurement of both sAPPα and sAPPβ is potentially useful for early diagnosis of dementia disorders.

Our results of statistical analyses clearly suggest that both sAPPs have good discriminatory power for the diagnosis of MCI-AD. Our data are partly consistent with previous reports. For example, multi-center studies by a single group detected significantly increased concentrations of sAPPα and sAPPβ in the CSF of AD and MCI-AD patients compared with controls and MCI-others patients [21, 24]. Alexopoulos et al. [22] reported that sAPPα and sAPPβ concentrations are higher in MCI patients than in AD patients. Perneczky et al. [25] reported increased sAPPβ levels in CSF of MCI-AD patients, and Alcolea et al. [26] showed that sAPPβ levels were increased in subjects with CSF evidence of AD pathophysiological processes among amnestic MCI and dementia patients; however, sAPPα was not measured in these studies. On the other hand, some other studies in which both sAPPα and sAPPβ were analyzed reported that neither was significantly different in AD or MCI-AD cases compared with controls [27–31]. Other recent studies in which only sAPPβ was measured showed that sAPPβ concentrations failed to distinguish between AD and healthy control groups [32, 33]. The reason why our study could detect significant increases in sAPPα and sAPPβ in MCI-AD and/or AD may be attributable to the accuracy of our assay method. The differences in assay kits as well as assay procedures could significantly affect the results of sAPPs measurement, as indicated by a recent validation study [34] as well as the current study. Our modifications made to the method to enhance its sensitivity possibly contributed to more accurate measurement of sAPPs concentrations. Further work is needed to optimize and standardize the assay methods of sAPPs in CSF.

We found that sAPPα and sAPPβ in CSF are highly correlated with each other, in good agreement with previous studies [20–23]. Intriguingly, we observed that sAPPα and sAPPβ are strongly correlated with p-tau (and total tau). This finding suggests that there may be pathological associations between tau and sAPPs, as the elevation of p-tau and total tau is thought to reflect the neurodegenerative changes associated with AD. There may also be physiological associations between tau and sAPPs, as tau is released into CSF under normal conditions [35, 36]. The elevated concentrations of sAPPβ in AD and MCI-AD patients could result from increased BACE1 processing of APP, considering that BACE1 protein levels or activities are increased in brains of AD as well as MCI patients [12, 37]. Consistently, some previous studies have reported a positive correlation between sAPPβ and total tau in MCI and AD cases [38] and preclinical AD subjects [39]. There is no clear explanation for the increase in sAPPα concentrations in MCI-AD subjects. However, it is possible that APP processing by α-secretase is increased in parallel with that by BACE1, which might be part of a protective response in the brain, as sAPPα has neuroprotective and neurotrophic activities [11, 12]. Other possibilities have also been suggested to account for the positive correlation between sAPPα and sAPPβ [22, 40]. We found no correlation between sAPPα and Aβ42, and only a weak negative correlation between sAPPβ and Aβ42, findings at least partly consistent with a previous report [20]. The decrease in Aβ42 in CSF is thought to be related to its accumulation and deposition, but not production, in the brain [41], which might explain the poor correlation between sAPPs and Aβ42.

Our study has several limitations. First, it is a small-scale study that does not include healthy control and MCI-others subjects; thus, the conclusions need to be replicated in additional studies with larger cohorts. It remains to be clarified whether sAPPs are useful in distinguishing the two MCI subgroups (MCI-AD and MCI-others); we will set out to investigate this issue in larger samples. It will also be of interest to determine whether sAPPs are altered at preclinical stages of AD. Second, because more than two facilities participated in this study, there could be some inadvertent bias in the measurements of p-tau and Aβ42. However, we consider that such bias is likely to be too small to affect the conclusions of this study. Third, because our study employs a cross-sectional design, disease stage-dependent changes in sAPPs need to be further explored in future longitudinal studies.

Together with advances in treatment strategies and diagnostic procedures, it has become increasingly important to accurately diagnose dementia disorders, including MCI, as early as possible. Specifically, differential diagnosis at the MCI stage or even in preclinical AD, is important for selecting patients for early therapeutic intervention. Although Aβ42 and p-tau (or total tau) are well-established biomarkers for AD-type dementia disorders, measuring CSF sAPPα and sAPPβ with high accuracy may provide a complementary approach for the early and precise diagnosis of patients with neurocognitive disorders.

## Conclusions

We here re-evaluated the value of CSF sAPPα and sAPPβ in the diagnosis of dementia disorders using a modified, sensitive detection method. Both sAPPα and sAPPβ were highly correlated with p-tau and total tau, suggesting that both sAPPs reflect neuropathological changes in the brain. sAPPα levels were specifically higher in the MCI-AD group compared with non-AD and control groups, and sAPPβ levels were higher in both AD and MCI-AD groups compared with other groups. Because both sAPPs have good discriminative power for the diagnosis of MCI-AD, we suggest that sAPPs in CSF are potentially useful and complementary biomarkers for early and accurate diagnosis of dementia disorders.

## Additional files

Additional file 1: Figure S1. Standard curves for measurement of sAPPα and sAPPβ concentrations. Typical standard curves for sAPPα and sAPPβ obtained using the original method (A) and those obtained using our modified method (B) are shown. The modified method yielded apparent differences, including higher values of absorbance at 450 nm at lower concentrations and the requirement for a much shorter time for the TMB reaction than the original method. (TIFF 325 kb)

Additional file 2: Figure S2. Correlations between total tau sAPPα or sAPPβ. Scatterplots show correlations between total tau and sAPPα (A) and between total tau and sAPPβ (B). (TIFF 300 kb)

Additional file 3: Figure S3. ROC curves demonstrating the discriminating ability of sAPPα (A), sAPPβ (B), and p-tau (C) in AD and MCI-AD versus other groups (non-AD and disease control). For sAPPα, area under the curve (AUC) = 0.736 [Asymptotic 95% Confidence Interval: 0.637–0.835] and the appropriate cut off value is sAPPα = 241, with sensitivity = 0.780 and specificity = 0.565. For sAPPβ, AUC = 0.766 [0.672–0.861] and the appropriate cut off value is sAPPβ = 436, with sensitivity = 0.780 and specificity = 0.630. For p-tau, AUC = 0.918 [0.864–0.972] and the appropriate cut off value is p-tau = 53, with sensitivity = 0.920 and specificity = 0.822. (TIFF 448 kb)

## Abbreviations

AD: Alzheimer’s disease
APP: Amyloid precursor protein
Aβ42: Amyloid β-ptotein 42
CSF: Cerebrospinal fluid
MCI: Mild cognitive impairment
MCI-AD: MCI due to AD
p-tau: Phosphorylated tau
sAPPα: Soluble APP-α
sAPPβ: Soluble APP-β
