# Supplementary figures and images for: Re-evaluation of soluble APP-α and APP-β in cerebrospinal fluid as potential biomarkers for early diagnosis of dementia disorders
Source: Biomark Res. 2017 Sep 22;5:28. doi: 10.1186/s40364-017-0108-5 (PMC5610422; doi:10.1186/s40364-017-0108-5)

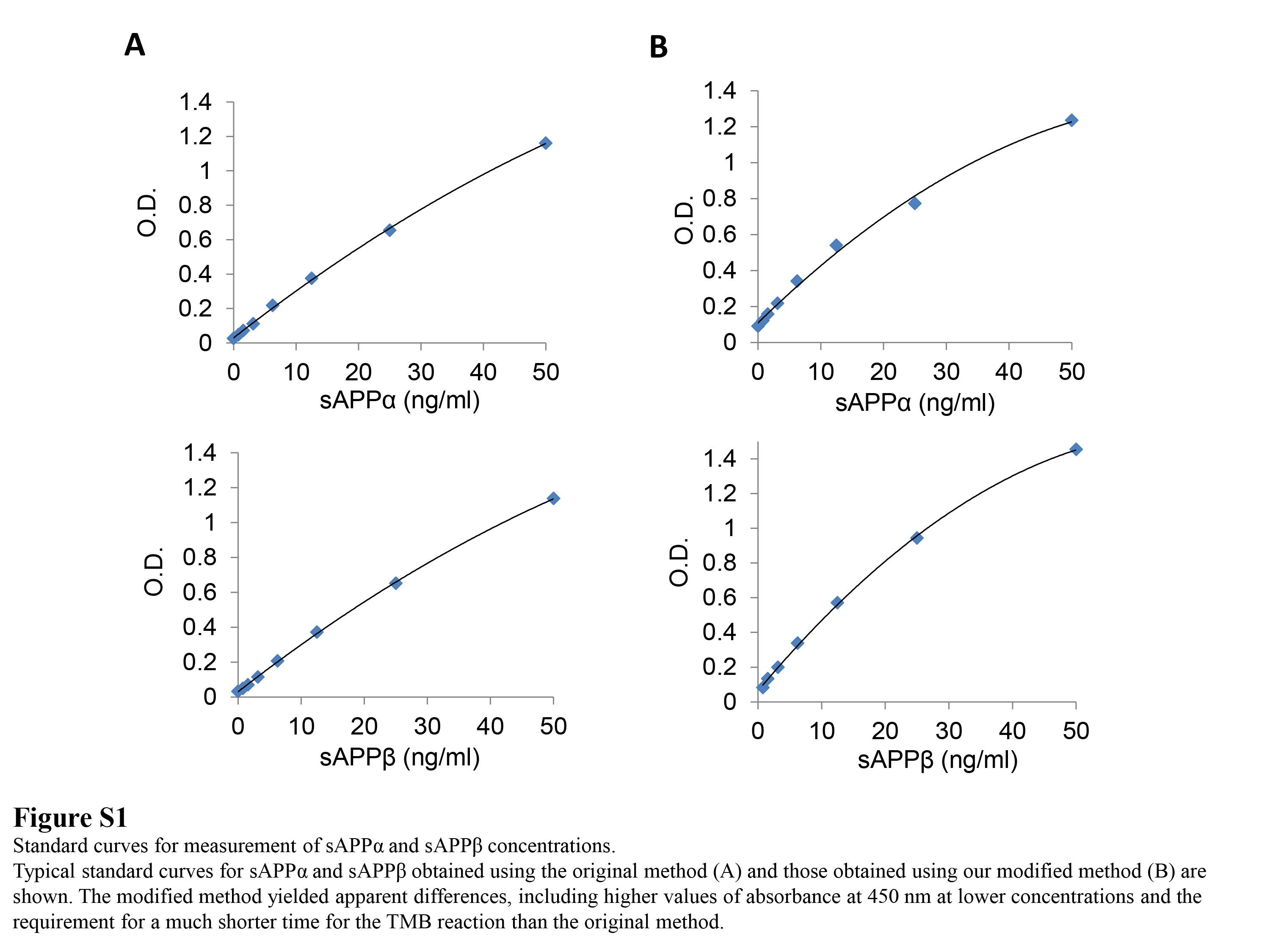

Supplement: Supplementary file 1 — Standard curves for measurement of sAPPα and sAPPβ concentrations. Typical standard curves for sAPPα and sAPPβ obtained using the original method (A) and those obtained using our modified method (B) are shown. The modified method yielded apparent differences, including higher values of absorbance at 450 nm at lower concentrations and the requirement for a much shorter time for the TMB reaction than the original method. (TIFF 325 kb) [file 40364_2017_108_MOESM1_ESM.tif]

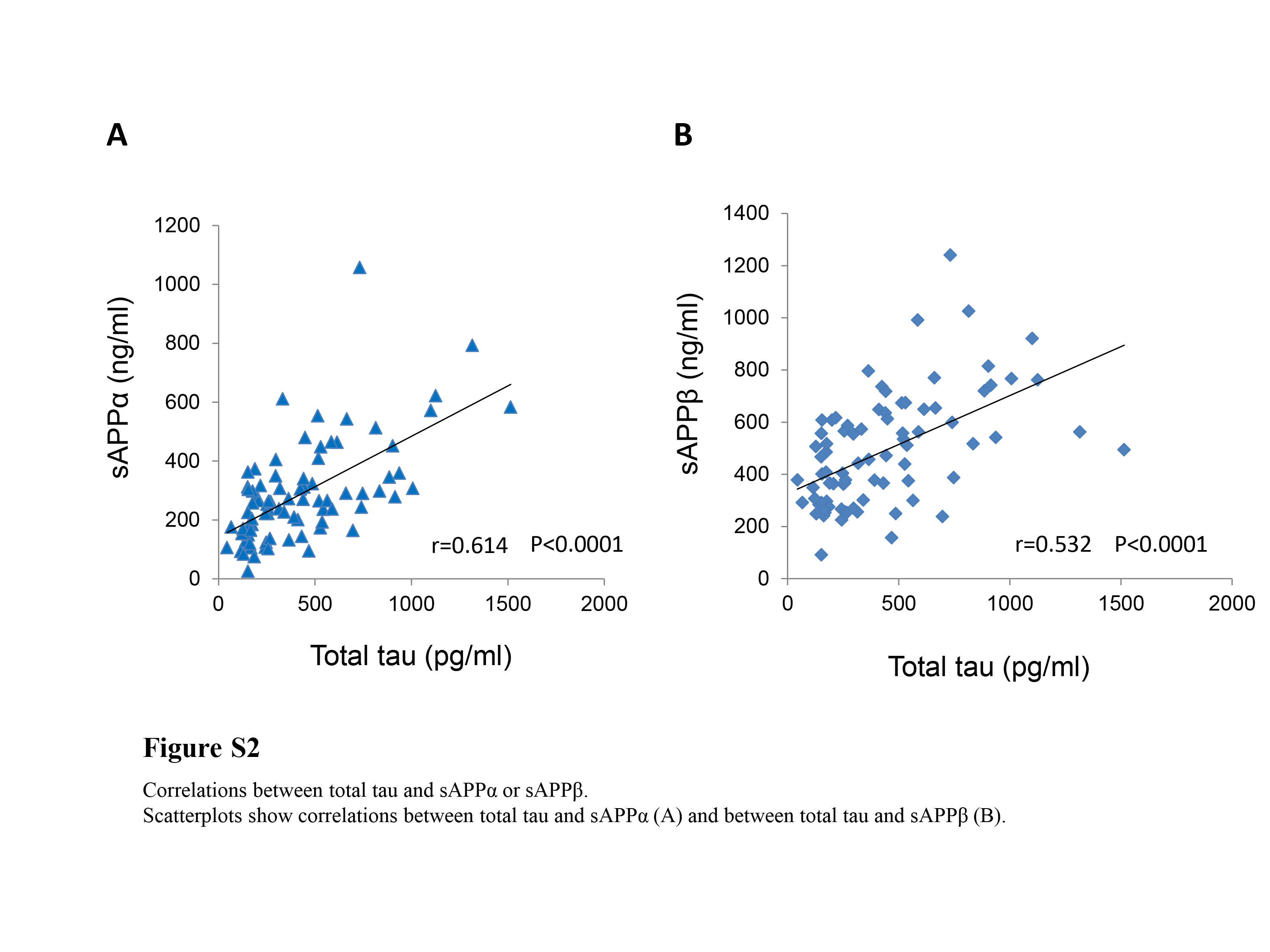

Supplement: Supplementary file 2 — Correlations between total tau sAPPα or sAPPβ. Scatterplots show correlations between total tau and sAPPα (A) and between total tau and sAPPβ (B). (TIFF 300 kb) [file 40364_2017_108_MOESM2_ESM.tif]

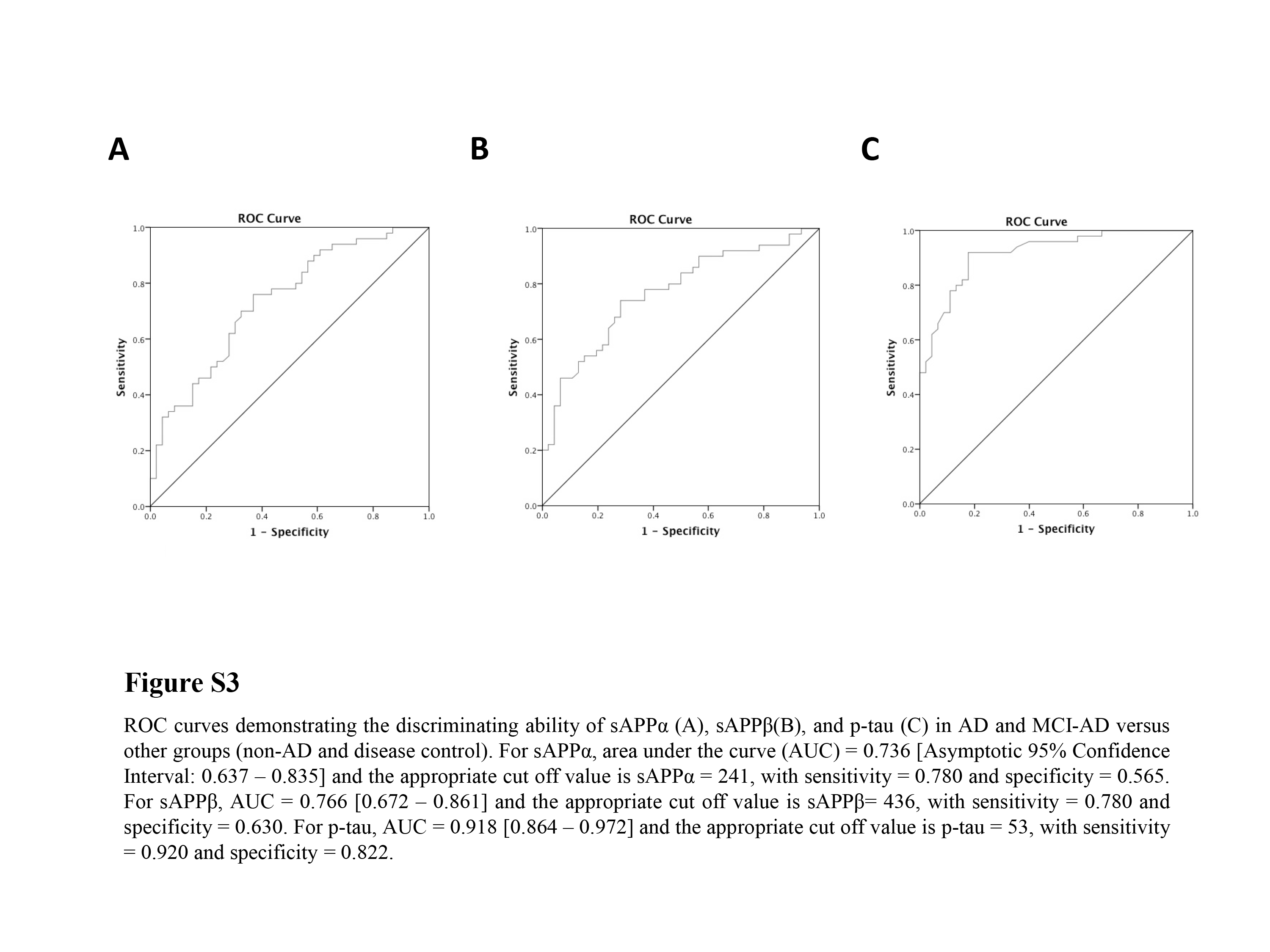

Supplement: Supplementary file 3 — ROC curves demonstrating the discriminating ability of sAPPα (A), sAPPβ (B), and p-tau (C) in AD and MCI-AD versus other groups (non-AD and disease control). For sAPPα, area under the curve (AUC) = 0.736 [Asymptotic 95% Confidence Interval: 0.637–0.835] and the appropriate cut off value is sAPPα = 241, with sensitivity = 0.780 and specificity = 0.565. For sAPPβ, AUC = 0.766 [0.672–0.861] and the appropriate cut off value is sAPPβ = 436, with sensitivity = 0.780 and specificity = 0.630. For p-tau, AUC = 0.918 [0.864–0.972] and the appropriate cut off value is p-tau = 53, with sensitivity = 0.920 and specificity = 0.822. (TIFF 448 kb) [file 40364_2017_108_MOESM3_ESM.tif]
